# Hepatitis E virus and fulminant hepatitis–a virus or host-specific pathology?

**DOI:** 10.1111/liv.12629

**Published:** 2014-07-28

**Authors:** Donald B Smith, Peter Simmonds

**Affiliations:** Centre for Immunity, Infection and Evolution, Ashworth Laboratories, University of EdinburghKing's Buildings, West Mains Road, Edinburgh, EH9 3JT, UK

**Keywords:** fulminant, hepatitis E virus, liver failure, virus variation

## Abstract

**Background & Aims:**

Fulminant hepatitis is a rare outcome of infection with hepatitis E virus. Several recent reports suggest that virus variation is an important determinant of disease progression. To critically examine the evidence that virus-specific factors underlie the development of fulminant hepatitis following hepatitis E virus infection.

**Methods:**

Published sequence information of hepatitis E virus isolates from patients with and without fulminant hepatitis was collected and analysed using statistical tests to identify associations between virus polymorphisms and disease outcome.

**Results:**

Fulminant hepatitis has been reported following infection with all four hepatitis E virus genotypes that infect humans comprising multiple phylogenetic lineages within genotypes 1, 3 and 4. Analysis of virus sequences from individuals infected by a common source did not detect any common substitutions associated with progression to fulminant hepatitis. Re-analysis of previously reported associations between virus substitutions and fulminant hepatitis suggests that these were probably the result of sampling biases.

**Conclusions:**

Host-specific factors rather than virus genotype, variants or specific substitutions appear to be responsible for the development of fulminant hepatitis.

The aetiology of hepatitis E virus infection (HEV) is complex. Many individuals exposed to HEV infection remain asymptomatic while others go on to develop an acute hepatitis of varying severity that usually resolves within 2–3 months [reviewed in (1)]. Chronic infection lasting a year or more has been observed rarely, and then only in immunocompromised individuals. Another potential outcome of HEV infection is fulminant hepatitis (FH), an uncommon rapidly deteriorating state with a poor prognosis involving pathologies such as hepatic encephalopathy, necrosis of hepatic parenchyma, coagulopathy, renal failure or coma.

Several previous studies have suggested that the pathogenicity of HEV infection might be genotype or strain dependent. For example, a bias towards genotype 4 has been noted in Japanese patients with FH or severe disease (2–4), while a recent study reports an association between infection with genotype 4 virus in France and higher levels of ALT and the presence of jaundice (5). There are also reports that disease severity (including FH) is associated with a particular strain of HEV genotype 3 (6), and that FH is associated with particular strains of genotype 1 (7) or genotype 4 virus (8). Further, several recent publications have suggested that there may be a link between particular substitutions in the HEV genome and the development of FH. For example, progression to FH has been associated with 142 synonymous and 8 nonsynonymous substitutions of genotype 1 virus (9), with the presence of two or three synonymous substitutions of genotype 4 virus (10,11), or with 12 unique amino acid substitutions in a genotype 4 virus from a FH patient (12).

However, an unacknowledged problem with some of these studies is the common geographical origin of the variants studied so the reported associations between particular virus substitutions and FH might simply occur because of epidemiological relationships amongst the viruses rather than because such substitutions were involved in the development of FH. This article re-examines published evidence for an association between FH and particular HEV genotypes, lineages or particular nucleotide substitutions.

## Materials and methods

Complete HEV genome sequences downloaded from Genbank were as follows: Genotype 1: JF443726, JF443725, JF443724, JF443723, JF443722, FJ457024, X98292, M73218, JF443721, JF443720, JF443719, JF443718, JF443717, AF459438, AF076239, X99441, AF051830, DQ459342, M80581, D10330, AF185822, L08816, D11092, D11093, L25595, AY204877, AY230202, AB720034, AB720035, JQ655734, AF444002, AF444003, L25547, M94177, Genotype 3: AB291955, Genotype 4 human isolates: AB291967, AB291959, AB193176, AB220971, AB220972, AB220973, AB091395, JQ740781, AB291966, AB291965, AB291968, AB220974, AB291964, AB220975, AB220976, AB220977, AB220978, AB220979, AJ272108, AB108537, AB097812, FJ763142, KC492825, AB698654, JQ655735, JQ655733, HQ634346, HM439284, AB369690, AB369688, AB197674, AB197673, AB193178, AB193177, AB099347, AB074917, AB074915, AB080575.

Sequences were aligned and annotated using SSE v1.1 (13) and phylogenetic analysis was performed using Mega 6 (14). Nucleotide positions were numbered relative to AB220978. The significance of associations between substitutions at each genome position and FH status was measured using Fisher's exact test using a significance level of *P* < 0.01 in a two-tailed test as implemented in an R script available upon request from the authors. The same data sets were analysed using meta-CATS (http://www.viprbrc.org) that uses both the chi-squared test of independence and Pearson's chi-squared test (15). The genotype 1 data set differed from that of (9) in that three identical or near-identical sequences (L25547, M94177 and AF444002) were removed, and three recently reported genotype 1 non-FH sequences (AB720034, AB720035 and JQ655734) were added.

## Results

### Is FH genotype-specific?

There are reports of patients developing FH after infection with genotype 1 (7,9,16,17), genotype 3 (6) and genotype 4 (8,10,12,18–22). A large outbreak of HEV genotype 2 in Namibia was associated with FH in 3/600 (0.5%) of individuals (23); although nucleotide sequences were not reported from the fulminant cases it seems likely that genotype 2 virus was involved. Hence, all four of the currently identified HEV genotypes known to infect humans can result in FH.

### Is FH strain-specific?

**Fig 1:**
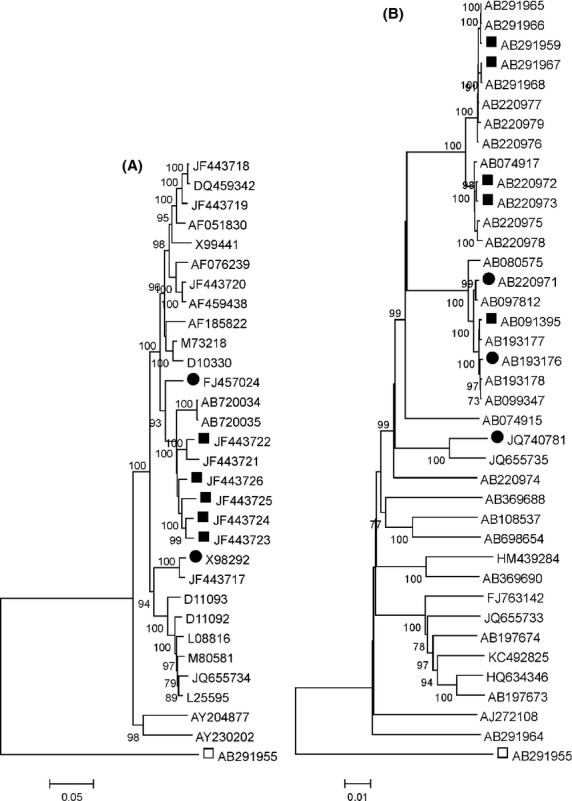
Phylogenetic analysis of complete HEV sequences from FH and non-FH patients. Distances between HEV genotype 1 (A) and genotype 4 (B) complete genome sequences are presented on a neighbor-joining tree (FH patients are indicated by symbols, ▪ for FH isolates from Pune or Hokkaido, and • for other FH isolates, □ for the genotype 3 outgroup). Branches supported by >70% of bootstrap replications (*n* = 500) are indicated.

We next investigated the possibility that FH results from the infection with particular strains of HEV. Phylogenetic analysis of the complete genome sequences of HEV derived from FH patients (Fig.1) reveals the presence of three lineages (groups of sequences supported by >70% of bootstrap replication) for both genotypes 1 and 4. Analysis of partial genome sequences of HEV from an additional 10 genotype 1 FH patients (EF015410, EF175962-4, EF206325&6 (7) and FJ230847-50), identified another genotype 1 lineage (Figure S1A, B), while analysis including the genotype 4 FH sequences (AB108659&60 (20), AB505793 (8), AB079762 (24) and AB114178) revealed a fourth genotype 4 lineage (Figure S1C–E). The ORF2 sequences of HEV genotype 3 FH isolates AB079763 (11), EF061404 and AB291955 (6) fell into three lineages (Figure S1F). Hence, these 31 FH-derived HEV variants comprise at least 11 lineages within genotypes 1, 3 and 4.

### Is FH transmissible?

Three published studies describe exposure from a common source (all HEV genotype 4) following which at least one individual developed FH. Of two men who consumed uncooked boar liver, one developed FH while the other individual had acute hepatitis (19). Similarly, of 13 individuals who had eaten grilled pig liver and intestines together, one developed FH and five seroconverted but were asymptomatic (21). One of these individuals transmitted HEV by blood transfusion to an individual who cleared the infection following interferon treatment. Finally, of 40 individuals who had eaten barbecued pig meat and entrails together, one developed FH, one had acute severe hepatitis, one had self-limited hepatitis, one seroconverted but had a subclinical infection, 10 were seronegative and the remainder were unavailable for follow up (22). These observations imply either that exposure to a particular strain of HEV is not sufficient to produce FH or that the penetrance of the trait is low. From the cases described above the penetrance would be 50%, 17% and 25%.

### Is FH associated with specific virus substitutions?

We tested possibility that particular substitutions are responsible for the development of FH by tabulating sequence differences in two of the common source outbreaks for which complete genome sequences were available (Table 1). None of the 17 sites at which viruses derived from the same source differed, were also variable in the other transmission set, and none were overrepresented amongst other FH isolates. Similarly, although a genotype 4 virus isolated from a FH patient had 12 unique amino acid substitutions (12), none of these substitutions were present in other genotype 4 FH sequences.

**Table 1:** FH Associations in common-source genotype 4 infections

Sequence		Nucleotide position																

		100	307	1963	2376	2968	3718	3737	3921	4072	5203	5377	5541	5637	6053	6412	6648	7006
		
Syn/Nsyn		Syn	Syn	Syn	NS	Syn	Syn	NS	NS	Syn	2/3	2/3	Syn	Syn	NS	NS	Syn	Syn
AB291959	FH	T	t	C	T	C	C	G	C	T	C	T	T	C	T	A	T	C
AB291965		c	t	t	T	C	t	a	C	c	t	T	T	t	c	A	c	t
AB291966		c	t	t	T	C	t	a	C	c	t	C	T	t	c	A	c	t

AB291967	FH	c	C	C	T	C	C	G	C	c	C	T	T	t	c	A	c	C
AB291968		c	t	C	a	t	C	G	t	c	C	T	c	t	c	g	c	C

AB193176	FH	c	t	C	T	t	C	G	C	c	C	T	T	C	c	A	T	C
AB220971	FH	c	t	C	T	t	C	G	C	c	C	T	T	C	c	A	T	C
AB220972	FH	c	t	C	T	C	C	a	C	c	t	T	T	t	c	A	c	C
AB220973	FH	c	t	C	T	C	C	a	C	c	t	T	T	t	c	A	c	C
AB091395	FH	c	t	C	T	t	C	G	C	c	C	T	T	C	c	A	T	C
JQ740781	FH	c	a	C	T	C	t	G	C	c	C	T	c	t	c	A	c	t
Other human genotype 4		T1	C3	C12	T23	C12	C18	G15	C25	T9	C18	T27	T18	C10	c27	A27	T16	C20
		c29	t21	t13	c4	a6	t8	a11	t2	c18	t9		c9	t11			c11	t7
			a1	a2		t6	g1							g4				
			g2			g3								a2				

Nucleotide substitutions present in a FH patient but not in a non-FH patient infected from the same source are capitalised. Nucleotides are numbered relative to AB220978; Syn, synonymous; NS, non-synonymous; 2/3 – overlap between ORF2 and ORF3 where substitutions are synonymous in one frame and non-synonymous in the other. Sequences derived from a common source of infection are enclosed between bold lines. The number of isolates with each nucleotide present amongst other non-FH genotype 4 sequences (*n* = 27) is indicated.

Previous studies have used statistical methods to identify substitutions that are associated with isolates derived from FH patients infected with HEV genotype 4 (10,11) or genotype 1 (9). However, interpretation of these results is complicated by the restricted geographical and temporal origin of the FH isolates; five of the seven genotype 1 FH isolates were from the single Indian city of Pune and were sampled in consecutive years (9) while five of eight FH-derived genotype 4 complete genome sequences were obtained on the Japanese island of Hokkaido from 2002 to 2006 (10,11). Our re-analysis of the genotype 1 data set using Fisher's exact test identified 110 sites significantly (*P* < 0.01) associated with FH, but 136 sites if only the five FH isolates from Pune were included, or four sites if only one of the Pune FH isolates was used. These four sites (positions 248, 946, 967 and 6312) were all synonymous, although position 6312 lies within a proposed secondary structure that is essential for virus replication (25). When we randomly assigned FH status to five of six non-FH isolates D11093, D11092, L08816, M80581, JQ655734 and L25595 that formed a well-defined lineage, >230 sites were detected. A three nucleotide deletion in ORF2/3 at position 5344 found in three FH isolates (9) was also present in two non-FH isolates, giving a non-significant association using Fisher's exact test (3/7 compared to 2/23, *P* = 0.06). Together, these observations suggest that the previous reports of sites significantly associated with FH amongst genotype 1 viruses may be actually reflect the common geographical and temporal origin of 5/7 FH isolates.

Similar re-analysis of genotype 4 complete genome sequences from FH (*n* = 8) and non-FH (*n* = 30) patients revealed only two sites significantly associated with FH (positions 1963 and 4795, both synonymous). These FH-associated substitutions were also present in >41% of the non-FH sequences and differed from the two sites reported from analysis of 22 isolates (10), or the eight sites reported from analysis of 28 isolates (11). Position 1963 differed between one FH and two non-FH isolates derived from a common source of infection (Table 1). Removing two or three of the most closely related Hokkaido sequences left no sites significantly associated with FH. The frequency of the double (synonymous) substitution U3148 and C5907 in FH isolates reached significance at the 5% level in Fisher's exact test (5/8 vs. 6/24, *P* = 0.031); a previous study reported a much lower *P* value of 0.0042 (11).

Repeating all these analyses using a different test for association, the Metadata-comparison analysis tool (meta-CATS, http://www.viprbrc.org) (15) that uses the chi-squared test of independence and Pearson's chi-squared test revealed fewer sites significantly associated with FH for both the full genotype 1 data set (45 significant sites compared to 110), and the full genotype 4 data set (0 significant sites compared to 2).

## Discussion

In contrast to several previous studies (6,7,9–12), we have been unable to identify specific HEV strains or genomic substitutions that are associated with FH, although the presence of synonymous substitutions at positions 3148 and 5907 (11) was significantly associated with FH at a reduced level of significance (5%). Progression to FH is not a genotype-specific property of HEV as all four of the genotypes currently known to infect humans have been associated with FH. In addition, FH is not a strain-specific property of particular lineages within each HEV genotype (Fig.1). Not all individuals infected from a common source develop FH and neither do particular substitutions appear to be associated with FH as no common mutations were observed in individuals with and without FH infected from a common source. Our re-analysis of the association between FH and substitutions at particular positions in the genome of genotype 1 and genotype 4 viruses suggests that previously reported significant associations may have been influenced by the restricted geographical and temporal sampling of FH isolates.

The identification of virus lineages or substitutions associated with the development of FH would be more difficult if this trait had incomplete penetrance. However, in this case, many more isolates from FH patients would be required. For example, a recent survey of virus determinants of FH following infection with hepatitis B virus that included 50 cases of FH with age- and sex-matched controls was able to demonstrate an association with the G1896A pre-core mutation (26), a substitution found in several other common source outbreaks in which there was a high frequency of FH (27). A different study identified two different substitutions (T1961 not T and C1962 not C) as significantly associated with FH (28). On the other hand, a study of 10 FH cases following acute infection with hepatitis A virus did not detect any difference in virus genotype between patient groups (29).

Although FH can develop following infection with any of the HEV genotypes known to infect humans, evidence that FH might be associated with particular genotypes comes from a survey of HEV in Japan over the last decade (30). Of 199 HEV patients, seven developed FH, these comprising 8.1% of those infected with genotype 4 but only 0.8% of those infected with genotype 3. However, the northerly island of Hokkaido which has <5% of Japan's population contributed 70% of the genotype 4 infections, and almost 70% of HEV infections there could be attributed to the consumption of uncooked pig liver, while in other regions most infections had no known source. Infection with HEV genotype 4 has also been associated with more severe disease (compared to genotype 3) amongst patients from France (5). In both cases, it is possible that the reported association between FH and HEV genotype 4 could reflect underlying epidemiological factors rather than a difference in virus pathogenicity. Experiments in animal models are currently limited to genotypes 1, 2 and 3 (31).

Alternatively, the development of FH consequent to HEV infection could be because of patient-specific factors. A precedent for this comes from previous studies showing a correlation between the severity of HEV infection (including FH) and pregnancy (32,33), pre-existing liver disease (2,34–37) and either a low (38) or high viral load (39). More detailed study of HEV infected FH patients from around the world should provide a definitive answer to the role of virus variation in this aspect of pathogenesis.

## Supporting information

**Figure S1.** Phylogenetic analysis of subgenomic HEV regions from FH and non-FH patients. Maximum composite likelihood distances between sequences are presented as neighbour joining trees for (A) genotype 1 (positions 4295–4601), (B) genotype 1 (4460–4786), (C) genotype 4 (123–448), (D) genotype 4 (4001–4821), (E) genotype 4 (5979–6390) and (F) genotype 3 (6034–6335). Sequences isolated from FH patients are indicated by • and the genotype 2 outgroup (M74506) by □. The percentage of bootstrap replicates supporting individual branches is indicated.

## References

[1] Kamar N, Bendall R, Legrand-Abravanel F (2012). Hepatitis E. Lancet.

[2] Mizuo H, Yazaki Y, Sugawara K (2005). Possible risk factors for the transmission of hepatitis E virus and for the severe form of hepatitis E acquired locally in Hokkaido, Japan. J Med Virol.

[3] Abe T, Aikawa T, Akahane Y (2006). Demographic, epidemiological, and virological characteristics of hepatitis E virus infections in Japan based on 254 human cases collected nationwide. Kanzo.

[4] Ohnishi S, Kang J-H, Maekubo H (2006). Comparison of clinical features of acute hepatitis caused by hepatitis E virus (HEV) genotypes 3 and 4 in Sapporo, Japan. Hepatol Res.

[5] Jeblaoui A, Haim-Boukobza S, Marchadier E, Mokhtari C, Roque-Afonso A-M (2013). Genotype 4 hepatitis e virus in france: an autochthonous infection with a more severe presentation. Clin Infect Dis.

[6] Takahashi K, Okamoto H, Abe N (2009). Virulent strain of hepatitis E virus genotype 3, Japan. Emerg Infect Dis.

[7] Pujhari SK, Kumar S, Ratho RK, Chawla YK, Chakraborti A (2010). Phylogenetic analysis and subtyping of acute and fulminant strains of hepatitis E virus isolates of North India with reference to disease severity. Arch Virol.

[8] Sugawara N, Yawata A, Takahashi K, Abe N, Arai M (2009). The third case of fulminant hepatitis associated with ‘Kitami/Abashiri strain’ of hepatitis E virus genotype 4. Kanzo.

[9] Mishra N, Walimbe AM, Arankalle VA, Hepatitis E (2013). Virus from India exhibits significant amino acid mutations in fulminant hepatic failure patients. Virus Genes.

[10] Inoue J, Nishizawa T, Takahashi M (2006). Analysis of the full-length genome of genotype 4 hepatitis E virus isolates from patients with fulminant or acute self-limited hepatitis E. J Med Virol.

[11] Inoue J, Takahashi M, Mizuo H (2009). Nucleotide substitutions of hepatitis E virus genomes associated with fulminant hepatitis and disease severity. Tohoku J Exp Med.

[12] Bu Q, Wang X, Wang L (2013). Hepatitis E virus genotype 4 isolated from a patient with liver failure: full-length sequence analysis showing potential determinants of virus pathogenesis. Arch Virol.

[13] Simmonds P (2012). SSE: a nucleotide and amino acid sequence analysis platform. BMC Res Notes.

[14] Tamura K, Stecher G, Peterson D, Filipski A, Kumar S (2013). MEGA6: molecular evolutionary genetics analysis version 6.0. Mol Biol Evol.

[15] Pickett BE, Liu M, Sadat EL (2013). Metadata-driven comparative analysis tool for sequences (meta-CATS): an automated process for identifying significant sequence variations that correlate with virus attributes. Virology.

[16] Donati MC, Fagan EA, Harrison TJ, Rizzetto M, Purcell RH, Gerin JL, Verme G Sequence analysis of full length HEV clones derived directly from human liver in fulminant hepatitis E. IX Triennial International Symposium on Viral Hepatitis and Liver Disease.

[17] Varma SPK, Kumar A, Kapur N (2011). Hepatitis E virus replication involves alternating negative- and positive-sense RNA synthesis. J Gen Virol.

[18] Takahashi K, Kang J-H, Ohnishi S (2003). Full-length sequences of six hepatitis E virus isolates of genotypes III and IV from patients with sporadic acute or fulminant hepatitis in Japan. Intervirology.

[19] Matsuda H, Okada K, Takahashi K, Mishiro S (2003). Severe hepatitis E virus infection after ingestion of uncooked liver from a wild boar. J Infect Dis.

[20] Yazaki Y, Mizuo H, Takahashi M (2003). Sporadic acute or fulminant hepatitis E in Hokkaido, Japan, may be food-borne, as suggested by the presence of hepatitis E virus in pig liver as food. J Gen Virol.

[21] Matsubayashi K, Kang J-H, Sakata H (2008). A case of transfusion-transmitted hepatitis E caused by blood from a donor infected with hepatitis E virus via zoonotic food-borne route. Transfusion.

[22] Miyashita K, Kang J-H, Saga A (2012). Three cases of acute or fulminant hepatitis E caused by ingestion of pork meat and entrails in Hokkaido, Japan: zoonotic food-borne transmission of hepatitis E virus and public health concerns. Hepatol Res.

[23] Maila HT, Bowyer SM, Swanepoel R (2004). Identification of a new strain of hepatitis E virus from an outbreak in Namibia in 1995. J Gen Virol.

[24] Suzuki K, Aiawa R, Okamoto H (2002). Fulminant hepatitis E in Japan — NEJM. N Engl J Med.

[25] Emerson SU, Nguyen HT, Torian U, Mather K, Firth AE (2013). An essential RNA element resides in a central region of hepatitis E virus ORF2. J Gen Virol.

[26] Kusakabe A, Tanaka Y, Mochida S (2009). Case-control study for the identification of virological factors associated with fulminant hepatitis B. Hepatol Res.

[27] Tong S, Li J, Wands JR, Wen Y (2013). Hepatitis B virus genetic variants: biological properties and clinical implications. Emerg Microbes Infect.

[28] Inoue J, Ueno Y, Kawamura K (2012). Association between S21 substitution in the core protein of hepatitis B virus and fulminant hepatitis. J Clin Virol.

[29] Hussain Z, Husain SA, Almajhdi FN, Kar P (2011). Immunological and molecular epidemiological characteristics of acute and fulminant viral hepatitis A. Virol J.

[30] Takahashi M, Okamoto H (2014). Features of hepatitis E virus infection in humans and animals in Japan. Hepatol Res.

[31] Purcell RH, Engle RE, Govindarajan S (2013). Pathobiology of hepatitis E: lessons learned from primate models. Emerg Microbes Infect.

[32] Jilani N, Das BC, Husain SA (2007). Hepatitis E virus infection and fulminant hepatic failure during pregnancy. J Gastroenterol Hepatol.

[33] Patra S (2007). Maternal and fetal outcomes in pregnant women with acute hepatitis E virus infection. Ann Intern Med.

[34] Hamid SS, Atiq M, Shehzad F (2002). Hepatitis E virus superinfection in patients with chronic liver disease. Hepatology.

[35] Ramachandran J, Eapen CE, Kang G (2004). Hepatitis E superinfection produces severe decompensation in patients with chronic liver disease. J Gastroenterol Hepatol.

[36] Acharya SK, Sharma PK, Singh R (2007). Hepatitis E virus (HEV) infection in patients with cirrhosis is associated with rapid decompensation and death. J Hepatol.

[37] Péron JM, Bureau C, Poirson H (2007). Fulminant liver failure from acute autochthonous hepatitis E in France: description of seven patients with acute hepatitis E and encephalopathy. J Viral Hepat.

[38] Saravanabalaji S, Tripathy AS, Dhoot RR (2009). Viral load, antibody titers and recombinant open reading frame 2 protein-induced TH1/TH2 cytokines and cellular immune responses in self-limiting and fulminant hepatitis E. Intervirology.

[39] Bose PD, Das BC, Kumar A (2011). High viral load and deregulation of the progesterone receptor signaling pathway: association with Hepatitis E-related poor pregnancy outcome. J Hepatol.

